# Genetic covariance between indices of body condition and immunocompetence in a passerine bird

**DOI:** 10.1186/1471-2148-5-61

**Published:** 2005-11-07

**Authors:** Deborah J Gleeson, Mark W Blows, Ian PF Owens

**Affiliations:** 1 School of Integrative Biology, University of Queensland, St Lucia, Brisbane Queensland 4072, Australia; 2 Division of Biology and NERC Centre for Population Biology, Imperial College London, Silwood Park, Ascot, Berkshire SL5 7PY, UK

## Abstract

**Background:**

Condition-dependence is a ubiquitous feature of animal life histories and has important implications for both natural and sexual selection. Mate choice, for instance, is typically based on condition-dependent signals. Theory predicts that one reason why condition-dependent signals may be special is that they allow females to scan for genes that confer high parasite resistance. Such explanations require a genetic link between immunocompetence and body condition, but existing evidence is limited to phenotypic associations. It remains unknown, therefore, whether females selecting males with good body condition simply obtain a healthy mate, or if they acquire genes for their offspring that confer high immunocompetence.

**Results:**

Here we use a cross-foster experimental design to partition the phenotypic covariance in indices of body condition and immunocompetence into genetic, maternal and environmental effects in a passerine bird, the zebra finch *Taeniopygia guttata*. We show that there is significant positive additive genetic covariance between an index of body condition and an index of cell-mediated immune response. In this case, genetic variance in the index of immune response explained 56% of the additive genetic variance in the index of body condition.

**Conclusion:**

Our results suggest that, in the context of sexual selection, females that assess males on the basis of condition-dependent signals may gain genes that confer high immunocompetence for their offspring. More generally, a genetic correlation between indices of body condition and imuunocompetence supports the hypothesis that parasite resistance may be an important target of natural selection. Additional work is now required to test whether genetic covariance exists among other aspects of both condition and immunocompetence.

## Background

Body condition is central to animal life histories because the expression of many traits critical to survival and reproductive success is condition-dependent [1,2]. Condition-dependence is, therefore, a topic of broad interest in both natural and sexual selection. One particularly striking example of the fundamental role of condition-dependence is in the context of mate choice [3-5]. Females often choose among males on the basis of condition-dependent signals, which honestly advertise male quality as the expression of these signals may trade-off with other life-history traits [6,7]. Many explanations have been put forward to explain the ubiquity of condition-dependent life histories and signals, with one influential theory predicting that the adaptive significance of condition-dependent signals may arise from the large number of genes that may influence variation in condition, thereby offering females the opportunity to assess a substantial proportion of male genomes in determining male quality [8]. Under this hypothesis, selection favours females who based their mate choice decisions on condition-dependent signals because such behaviour increases the females' chances of obtaining good genes for their offspring. This line of reasoning can be extended to predict that one class of genes that may be of particular interest to females are those loci that contribute to variation in parasite resistance [9], a major determinant of reproductive success and survival in many species [10]. The condition-mediated immunocompetence-handicap hypothesis (CMIH) [7,11-14], proposes that females base their mate choice decisions on condition-dependent male signals in order to obtain genes that confer high immunocompetence for their offspring.

A key requirement of the CMIH hypothesis, and other related life history hypotheses [15-19], is the presence of positive genetic covariance between body condition and immune response. The CMIH hypothesis proposes that it is this genetic covariance that enables a condition-dependent signal to advertise the quality of the parasite resistance genes that a male carries [14]. Although there is abundant evidence for positive phenotypic associations between body condition and immunocompetence [12,15,16,18,20-22], phenotypic analyses are insufficient to validate the CMIH hypothesis because it remains unknown whether females selecting males with good body condition simply obtain a healthy mate, or if they actually acquire genes for their offspring that confer high immunocompetence. An additive genetic component has been established in several experimental systems for both body condition [[23], but see [24]] and immune response [[25-29]; but see [30-32]]. There is also evidence of a genetic correlation between immune function and sexual signals [33,34], between immune function and life history traits [35] and between body condition and male signal [36]. As far as we are aware, however, it has not been empirically demonstrated that variation in immune response is mediated by genetic variation in body condition, a key element of the CMIH hypothesis [14].

The overall aim of this study was to test directly for genetic covariance between indices of body condition and immunocompetence in a small passerine bird, the zebra finch *Taeniopygia guttata*. Zebra finches provide an ideal opportunity to determine if this critical genetic association exists for two reasons. First, this species is a model system for the study of sexual selection, in which female choice is based on a number of condition-dependent male signals that include song rate and bill colour [37-40]. Second, there is phenotypic evidence of condition-dependent expression of immunocompetence in this species [4,16]. We therefore used this system to investigate the genetic basis of covariation between an index of body condition and an index of immunocompetence using a cross-fostering experiment. Here we implement the cross-fostering experimental design of Riska et al. [42] to estimate additive genetic components of variance. An important advantage of this method was that it allowed us to partition the genetic covariance between these traits into sources attributable to direct additive genetic covariance, additive maternal genetic covariance, and the covariance between these sources.

## Results

### Genotype-environment interaction

**Figure 1:**
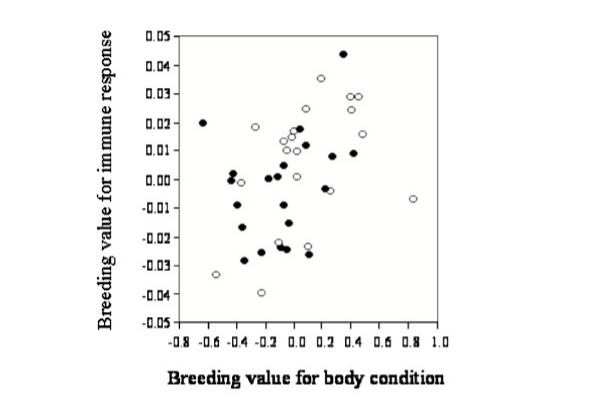
**Genetic correlation between our indices of immune response and body condition**. Each point represents the average breeding value across a single breeding pair. Filled and hollow circles show breeding pairs derived from the Alice Springs and Townsville sites respectively. Breeding values were estimated as BLUPs from the linear model described in Equation (1) in the Methods section.

The interaction term (***I***_*ijk*_) testing for a genotype-environmental interaction for Equation (1) was not significant for our indices of either immune response (*F*_*19,42 *_= 0.86; *p *= 0.63) or body condition (*F*_*19,42 *_= 0.81, *p *= 0.69). This showed that chicks from the two sampling sites did not respond differently to nest environments from the other population. Similarly, there were no significant differences between the sites for the phenotypic means of our indices of either immunocompetence (*F*_*1,119 *_= 0.88, *p *= 0.35) or body condition (*F*_*1,119 *_= 3.28, *p *= 0.08), or the breeding values of broods for either trait (Immunocompetence *F*_*1,39 *_= 1.85, *p *= 0.18: Body condition *F*_*1,39 *_= 3.32, *p *= 0.08; Figure 1). As explained in the Methods section, the marginally non-significant difference between the populations in our index of body condition is not sufficient to cause a spurious correlation between our indices of body condition and immunocompetence. As shown in Figure 1, although one population tends to have slightly higher breeding values for our index of body condition, both populations span almost the full range of values for both traits. Site was not, therefore, considered in the subsequent genetic models.

### Genetic covariance

When we based our analyses on comparisons between siblings alone, we detected positive genetic covariance between our indices of immune response and body condition. A plot of the breeding values, based on BLUP methodology, for our indices of immune response and body condition visually demonstrates the positive trend indicating a positive genetic correlation (Fig. 1).

The mixed-model approach detected positive covariances for observational components of covariance y_1 _and y_3 _for our indices of both immune response and body condition (Table 1). These components accounted for the majority of additive genetic variation in the design matrix (Table 2), which was relatively free of confounding non-additive and environmental effects. The estimates of additive direct genetic variance for our indices of immune response and body condition were 0.034 ± + 0.016 (p = 0.036) and 0.149 ± + 0.156 (p = 0.342), respectively. Most importantly, however, we detected significant positive genetic covariance (0.053 ± + 0.024, p = 0.030) between our indices of immunocompetence and body condition (Table 3). The estimate of the genetic correlation (*r*_*A*_) between our indices of immune response level and body condition was 0.75 ± + 0.41 (Table 3), suggesting that approximately 56% of the additive genetic variance in body condition can be explained by genetic variation in our index of immune response, which was calculated as square of the genetic correlation [43]. No other causal components were significant in any of the analyses.

**Table 1:** Observed components of variance for ten types of relatives.

Observed component (y_i_)	Method and types of relatives used to obtain component	Immune response	Body condition	Covariance
y_1_	covariance between sire and offspring	0.003	0.106	0.019
y_2_	covariance between nurse and offspring where the nurse is not the genetic dam	0.002	-0.122	-0.013
y_3_	covariance between dam and offspring where the offspring was nursed by an unrelated dam	0.01	0.119	0.002
y_4_	covariance between dam and offspring, where the dam is also the nurse.	0.006	0.022	0.022
y_5_	covariance between full sibs raised by different nurses	0.010	0.328	0.025
y_6_	covariance between unrelated sibs where the offspring were nursed by an unrelated dam	0.225	0.179	0.066
y_7_	covariance between full sibs raised by different nurses	0.002	0.239	-0.002
y_8_	covariance between unrelated sibs raised the same nurse	0.002	0.076	0.012
Y_9_	covariance between unrelated sibs, each nursed by the genetic dam of the other	-0.215	0.149	-0.042
Y_10_	variance among full sibs all with the same nurse	0.031	0.609	0.011

Definitions modified from Riska et al. [42]. Further details are in the Methods.

**Table 2:** Design matrix (X) displaying theoretical causal components of observed variances and covariances.

Observed component	Causal components					
	
	σ^2^_AO_	σ^2^_DO_	σ_AOAM_	σ^2^_AM_	σ^2^_DM+C_	σ^2^_E_
Y_1_	0.5	0	0.25	0	0	0
Y_2_	0	0	0.75	0.5	0	0
Y_3_	0.5	0	0.25	0	0	0
Y_4_	0.5	0	1.25	0.5	0	0
Y_5_	0.5	0.25	1	1	1	0
Y_6_	0.5	0.25	0	1	1	0
Y_7_	0.5	0.25	0.5	0	0	0
Y_8_	0	0	0.5	1	1	0
Y_9_	0	0	1	0	0	0
Y_10_	0.5	0.75	0	0	0	1

σ_AO_^2 ^= additive direct genetic variance, σ_DO_^2 ^= dominance direct genetic variance, σ_AOAM _= direct-maternal additive genetic covariance, σ_AM_^2 ^= additive maternal (or 'indirect') genetic variance, σ^2^_DM+C _= dominance maternal genetic variance and common environmental variance (maternal and cage), and σ_E_^2 ^= residual environmental variance. Further details are in the Methods.

**Table 3:** Genetic and non-genetic sources of variance and covariance.

source of variation	immune response b_i _± SE	body condition b_i _± SE	covariance b_i _± SE
σ_AO_^2^	0.034 ± 0.016*	0.149 ± 0.156	0.053 ± 0.024*
σ_DO_^2^	0.069 ± 0.062	0.386 ± 0.526	0.010 ± 0.110
σ_AOAM_	-0.044 ± 0.026	0.142 ± 0.178	-0.035 ± 0.029
σ_AM_^2^	0.079 ± 0.046	-0.457 ± 0.346	0.051 ± 0.055
σ^2^_DM+C_	-0.055 ± 0.033	0.465 ± 0.327	-0.018 ± 0.049
σ_E_^2^	-0.038 ± 0.049	0.245 ± 0.385	-0.023 ± 0.080
σ_P_^2^	0.045 ± 0.101	0.929 ± 0.841	0.037 ± 0.159

σ_AO_^2 ^= additive direct genetic variance, σ_DO_^2 ^= dominance direct genetic variance, σ_AOAM _= direct-maternal additive genetic covariance, σ_AM_^2 ^= additive maternal (or 'indirect') genetic variance, σ^2^_DM+C _= dominance maternal genetic variance and common environmental variance (maternal and cage), and σ_E_^2 ^= residual environmental variance. Asterisks denote statistical significance (* p < 0.05)

## Discussion

The relative roles of genetic and non-genetic factors in determining immunocompetence in birds is controversial. Although theoretical models of sexual selection tend to assume that such traits have a high genetic component, previous empirical evidence has often proven equivocal. The results of our cross-fostering experiment support claims that a part of the variation in at least one aspect of immune response is caused by genes [25-29]. Since males therefore differ in genetically-based levels of this aspect of immunocompetence, females could conceivably target immunocompetence during mate choice as predicted by the CMIH hypothesis.

In addition to indicating that immunocompetence is heritable in zebra finches, our experiment found substantial positive genetic covariance between our indices of immunocompetence and body condition. We found that approximately 56% of genetic variation in our index of body condition may be explained by genetic variation in our index of immune response. Since secondary sexual ornaments are typically condition-dependent in zebra finches [39,40], females that select males on the basis of well developed ornaments are likely to gain genes for their offspring which confer higher levels of immunocompetence. Therefore, our findings are consistent with the hypothesis that the genes that determine parasite-resistance may be a major target of sexual selection in this species.

The significant genetic covariance between our indices of immunocompetence and condition implies that the same genes underlie a proportion of the variation in both traits. However, although females selecting males with well developed ornaments are likely to gain genes that confer higher levels of immunocompetence, our genetic analysis is not sufficient to conclusively demonstrate that there are some genes that affect our indices of both immunocompetence and body condition. It is possible that linkage disequilibrium, generated by selection for both traits, may also contribute to the genetic covariance we have found between these two traits. Distinguishing pleiotropy from linkage disequilibrium in such a species is difficult; one approach would be the development of a pleiotropic quantitative trait loci map of both traits, but such techniques are yet to be applied in wild passerine populations.

The magnitude of the genetic correlation between our indices of immunocompetence and condition is surprising because theory predicts that such heritable genetic variation should be eroded through selection. What factors could maintain such variation? One possible explanation is that a third trait not included in this analysis trades off with our indices of immunocompetence and condition [44]. For example, one aspect of growth rate displays a negative genetic correlation with our index of immunocompetence (DJG unpublished data), suggesting that a more complex model of resource allocation than the simpler two-trait system of our indices of immunocompetence and condition might need to be considered to understand the maintenance of genetic variance in these traits.

Our results should be interpreted cautiously as this study suffers from a number of limitations. First, although our reciprocal cross-fostering design is efficient at detecting additive genetic effects, it is less powerful in estimating other quantitative genetic components. Our results indicated that none of the other causal components of covariance that we estimated were found to be significant. On first inspection, this suggests that non-additive genetic and environment effects do not play a role in generating covariance between our indices of immunocompetence and body condition, but we caution that this interpretation would be premature. Because the cross-fostering experimental design and the subsequent analytical method that we have used are primarily designed to detect additive genetic components of variance and covariance [42], we cannot exclude the possibility that environmental and/or non-additive components do exist and that we have simply failed to detect to them. This possibility is highlighted by the covariance estimates in Table 3, many of which are large in magnitude and are only non-significant because of the very large standard errors. Under these circumstances, no firm conclusions about the absence of environmental or non-additive covariance between these two traits can be drawn.

A second limitation of cross-fostering designs that use full sib cross-fostering is that they can only control for those aspects of environmental variance that occur after the cross-foster manipulation itself. In the case of avian studies, chicks are typically cross-fostered within 48 hours of hatching. Hence, although such studies can estimate variation associated with later incubation and parental feeding, they cannot deal with variance in factors such as the way mothers provision eggs or anything that happens in first few hours in the nest. There is always a risk, therefore, that cross-foster studies will inflate the estimate genetic components because these also include pre-cross-foster environmental components. Nevertheless, in the case of our study such environmental covariance is unlikely to be solely responsible for the high positive genetic covariance between our indices of immunocompetence and condition because the sire-offspring observation component is large and positive, which is unaffected by this source of variation.

The third limitation of our study is the lack of information about the adults used to establish the breeding experiment. Because the adults were caught from wild populations we do not know whether they were related to one another, their immunological history, or whether the parental generation experienced selection, all of which could effect the pattern of variation among individuals. In addition, although field parent and laboratory offspring relationships have been used to estimate genetic components of variance, it is not clear how differences between the lab and field environments would affect the genetic estimates in a model as complex as the one employed here.

Finally, our study is also limited by the fact that we have only used a single index of body condition and a single index of immunocompetence. Using residual values of body weight on skeletal size is a widely used index of body condition in birds, but it is well established that this method is not without its limitations and dangers [24,45]. Such an index cannot, for instance, differentiate between different aspects of condition, such as fat deposition and muscle size, and it has been shown that such an index can retain an element of body size itself. We nevertheless used this measure for our genetic tests, firstly, because it the index of body condition that has been used widely in previous phenotypic studies and is therefore of particular interest to avian studies, secondly, because it has repeatedly been shown to be under selection in avian populations [46], and thirdly, because it is not currently possible to perform multivariate analyses (with body size and tarsus length as separate covariates) using the genetic framework employed here. It would nevertheless be interesting in the future to test for genetic correlations among alternative measures of body composition, and to perform multivariate analyses if the statistical techniques are developed. Similarly, like any other index of immune response, our single measure of cell-mediate immunity cannot provide information on all elements of vertebrate immunocompetence [18,47]. Again, we used this index in our genetic study because it has been widely employed in phenotypic tests [15,20,22,30,40], has been shown to be associated with important components of fitness such as survival [20,30], and there is even limited evidence that variation in this measure may be positive associated with variation humoral immune response [[48], but see [20]]. But this should not obscure the facts that future studies of other elements of immunity are required to obtain a comprehensive understanding of the genetic basis of parasite resistance, and that multivariate models would help to tease these apart. Ideally, a full genetic variance-covariance matrix is required for a series of indices of body condition and a series of indices of immunocompetence, but this is well beyond the scope of the current study.

## Conclusion

Our results support a key prediction of the CMIH hypothesis; that there is positive genetic covariance between an index of body condition and an index of immunocompetence. More generally, although we have primarily been concerned here with the link between our indices of immunocompetence and body condition in the context of the CMIH hypothesis, a genetic link between indices of condition and immune function would also have implications outside sexual selection theory. Condition-dependence is a general feature of many aspects of life histories in many animal species [1,2]. The genetic correlation between our indices of immunocompetence and body condition that we have found in zebra finches suggests that the inter-relationships between many such traits may also prove to be parasite-mediated [12,19]. The prevalence of condition-dependent life histories may therefore arise, in part at least, through parasite resistance being a target of both natural and sexual selection. The ongoing challenge is to test the generality of these findings, that is whether there is significant positive genetic covariance between other indices of body condition and immunocompetence. More generally, it would be interesting to know the pattern of genetic covariance between various measures of condition and a suite of fitness-related traits.

## Methods

### Populations and experimental design

To sample the genetic variance present in field populations, we caught eighty zebra finches from the wild and then conducted a partial cross-fostering experiment [42,49], under standardised laboratory conditions. Zebra finches were caught between August 1998 and February 1999 using mist nets at sites near Alice Springs and Townsville, Australia. Twenty birds of each sex were collected from each site, and transported by plane. For the breeding experiments, birds were kept outdoors in two large free-flight breeding aviaries at the University of Queensland. Each aviary housed twenty pairs of finches, which were introduced at the same time and allowed to form pairs naturally. All pairs bred during the course of this experiment. Ad libitum food and water were provided during the study period, including fresh green material. Birds from the two collection sites were housed separately, but offspring were cross-fostered between aviaries.

A reciprocal partial cross-fostering design was used to maximise the opportunity to estimate maternal and non-additive genetic components of variance [42]. For each reciprocal cross-foster event, the two nests between which chicks were transferred was referred to as a 'block' of nests. Broods typically consisted of four offspring, with two offspring being transferred between a pair broods. Broods within a block were matched with respect to hatching date and clutch size. Chicks were selected randomly for cross-fostering except for 'runts', which were not cross-fostered and always died before measurement. The identity of individual chicks was monitored by clipping their downy head-tufts until they were large enough to carry individually-numbered leg bands. Offspring were cross-fostered immediately after hatching, and body condition and immune response were measured 17 days later.

### Measurement of traits

The element of immunocompetence that we measured was experimentally-induced T-lymphocyte cell-mediated immune response, an acquired component of the avian immune system[18]. We induced a cell mediated immune response through intradermal injection of phytohemagglutinin-P [15]. For each bird, 0.1 mg of phytohemagglutinin-P in 0.02 ml of phosphate buffered saline was intradermally injecting the right wing web, with 0.02 ml of phosphate buffered saline being injected into the opposite wing web as a control. The thickness of each wing web was measured at the injection site both immediately before and 24 hours after the injections. Twenty four hours is the standard reaction period in avian studies and is the point at which the swelling is typically maximum [15]. Measurements were taken three times to the nearest 0.001 mm using a digital micrometer and 'before' and 'after' averages were calculated for each wing. We then calculated the swelling for each wing, which was the difference between the 'before' and 'after' averages. Finally, cell-mediated immune response was calculated for each individual as the difference in swelling between the phytohemagglutinin-P injected wing and the control wing.

In this study body condition is defined as, and was measured as, the residual value from the regression of body mass on tarsus length [46]. To enhance the clarity of our writing we refer to this measure throughout this study as an index of body condition, although the reader should keep in mind that this index is derived from an estimate of residual body mass. Body mass was measured to 0.01 g using a Petit Precision balance (model MK-200 200 g × 0.01 g). Tarsus was measured to the nearest 0.5 mm using digital callipers. For parental individuals, all measurements were taken immediately before they began a breeding cycle.

### Genotype-environment interactions

Because we collected birds from two different sites, we tested for possible genotype-environment interactions for our indices of both immune response and body condition. A two-way factorial ANOVA comprising nest of origin, nest of rearing and the interaction term for each block of nests was used [49-51]:

***Z***_***ijkl ***_= **μ **+ ***P***_***i ***_+ ***M***_***ij ***_+ ***N***_***ik ***_+ ***I***_***ijk ***_+ ***e***_***ijkl ***_    (1)

where, ***P***_***i ***_= average effect of the *i*th cross-fostered block of nests, ***M***_***ij ***_= direct effect of the *j*th (genetic) mother within the *i*th block (*j *= 1 or 2), ***N***_***ik ***_= *k*th (unrelated) nurse within the *i*th block (*k *= 1 or 2), ***I***_***ijk ***_= *M *× *N *interaction within the *i*th block, and ***e***_***ijkl ***_= residual error for the *i*th offspring of the *j*th mother raised by the *k*th nurse within the *l*th block of nests.

The ***I***_***ijk ***_term of Eq. **1 **tested for the presence of a genotype-environment interaction in this experiment as nest of origin also represented genetic population of origin and nest of rearing also represented the population of rearing. Significance of nest of origin (***M***_***ij***_) and nest of rearing (***N***_***ik***_) was tested using the interaction term (***I***_***ijk***_) as the error, type III sums of squares for unbalanced designs. In all genetic models genetic relationships were inferred on the basis of the male and female providing care at the nest in question, as extra-pair paternity in zebra finch colonies is low (2.4% of chicks) [52].

When using individuals from two different populations there is also the risk that such populations could differ with respect to the parameters under study. Specifically, if the populations differed with respect to both traits then pooling individuals from the two populations might generate spurious covariance between the traits. It is important to note here that the populations need to differ for both traits and not just one of them. This is because, if populations only differ with respect to in one variable, this would just increase variation along a single axis. To assess these possibilities we therefore tested for differences between the source populations in both the phenotypic means and breeding values of our indices of both immune response and body condition.

### Genetic covariance

We used two related methods to test for a genetic correlation between our indices of immunocompetence and body condition. To facilitate comparisons with other studies, we first used the standard method for analysing cross-foster experiments, which is based on using offspring values alone [49]. We used BLUP methodology to calculate breeding values [49] and plotted these against each other to visualise the pattern of covariance. However, analyses based on full-siblings alone may lead to biased estimates of additive genetic components since pre-fostering maternal effects and dominance genetic variance cannot be partitioned out from additive genetic effects [49,53]. The confounding of genetic and environmental factors may be of particular concern in birds, where the egg environment may provide a source of direct maternal effects [20].

To avoid the potential problems associated with genetic estimates based on comparisons between full-sibs alone, we used an under-utilised second method based on a mixed model [42] to test for a genetic correlation. An important advantage of this type of model is that it allows both offspring and parental trait values to be used to distinguish a number of sources of variation, thereby enabling the separation of additive genetic variance from dominance genetic variance and direct maternal effects [42,49]. The degree of similarity between ten types of relatives (Table 1) was then used to estimate six genetic and non-genetic causal components contained in each of these observational components, which are displayed in the design matrix **X **(Table 2).

The observation vector **y **comprised ten observational components of variance (y_i_) that were estimated by the various methods listed in Table 1. All components were computed using methodology taken directly from Riska et al. [42], with the exception of y_9_. The estimation of the direct-maternal additive genetic covariance which is isolated by observational component 9 has been the source of some confusion in the literature. Rutledge et al [54] first proposed σ_AOAM _could be estimated using the interaction term in (1), which more recently was also advocated as an appropriate way of estimation in Lynch and Walsh (1998). However, this method of estimation was subsequently shown to be incorrect [55,56]. Riska et al [42] outlined that component 9 could be estimated in another two equivalent ways, but we did not find the exact numerical agreement between these two methods suggested by these authors (unpublished results). We therefore used an established alternative method for estimating component 9 described by [55,57], which uses the difference between the covariance between full sibs raised by different nurses, and the covariance between unrelated sibs raised by the same nurse. We note that a limitation of the Riska et al [42] approach is that using the same mean squares for the estimation of different causal components generates covariance between the estimates which is not accounted for in the model as implemented either by Riska et al [42,55] or here (i.e. the off-diagonal elements of the **V **matrix are set to zero, see below). By using the estimation method of component 9 employed here, this potential problem is likely to be exacerbated as the estimate of component 9 is a linear combination of the mean squares used in other observational components (5 and 6). Nevertheless, component 9 as estimated here has an established interpretation, and facilitated the isolation of the important σ_AOAM _causal component.

Variances of the observational components were used as the diagonal elements of the square matrix **V**, with off-diagonal elements all zero. To obtain variances for each observational component [49] for components 1–4:

*VAR*(σ^2^) = (*VAR*(*A*) *VAR*(*B*) + *COV*(*A*, *B*)^2^) / (*N*)     (2)

where *A *and *B *represented the two kinds of individuals whose covariance is being estimated and *N *is the number of bivariate observations. The variance of components 5–10 were estimated as weighted sums of the variances of the appropriate mean squares, where the variance of a mean square is given by (Ref [49], equ. A1.10c):

*VAR*(σ^2^) = (2*MS*^2^) / (*N *+ 2)     (3)

in which *MS *represents the mean square of the term of interest and *N *is the number of blocks. The causal components of variance were estimated as elements of the vector:

***b ***= (**X'V**^-1^**X**)^-1^**X'V**^-1^***y ***    (4)

with covariance matrix:

**S **= (**X'V**^-1^**X**)^-1^.     (5)

where the square root of the corresponding diagonal element of **S **was used to estimate the standard error of ***b***. Phenotypic variance was estimated as the sum of the elements of ***b ***and its corresponding standard error approximated by the square root of the summed diagonal elements of **S**.

Estimation of the genetic correlation between our indices of body condition and cell-mediated immune response level required all observational components to be estimated as cross-covariances [58]. Cross-covariances were estimated as the product of the values of our indices of body condition and immune response for each individual and the sums of products partitioned according to the source of variation (Falconer 1981). The variances of these cross-covariances (used as the diagonal elements of the square matrix **V**) were determined by calculating separate variances of cross-covariances for (i) our index of body condition in parents and our index of immune response in offspring, and (ii) our index of immune response in parents and our index of body condition in offspring. The mean was then taken of these two variances of cross-covariances. The additive genetic correlation (*r*_*A*_) and an estimate of its standard deviation were calculated using equations 19.2 and 19.4 in Falconer [58], respectively. The proportion of additive genetic variance in our index of body condition explained by genetic variation in our index of immune response was then calculated as the square of the genetic correlation [43].

## Authors' contributions

DJG helped to design the project, collected animals from the wild, conducted all crosses and measurements, performed statistical analyses, and helped to write the paper. MWB helped with the statistical analyses and the writing of the paper. IPFO helped to design the project, collect animals, and write the paper. All authors read and commented on drafts of the manuscript, and approved the final manuscript.
